# Molecular characterization of breast cancer cell lines through multiple omic approaches

**DOI:** 10.1186/s13058-017-0855-0

**Published:** 2017-06-05

**Authors:** Shari E. Smith, Paul Mellor, Alison K. Ward, Stephanie Kendall, Megan McDonald, Frederick S. Vizeacoumar, Franco J. Vizeacoumar, Scott Napper, Deborah H. Anderson

**Affiliations:** 1 Cancer Cluster, University of Saskatchewan, 107 Wiggins Road, Saskatoon, SK S7N 5E5 Canada; 2 Vaccine Infectious Disease Organization - International Vaccine Centre (VIDO-InterVac), University of Saskatchewan, 120 Veterinary Road, Saskatoon, SK S7N 5E3 Canada; 3 Cancer Research, Saskatchewan Cancer Agency, 107 Wiggins Road, Saskatoon, SK S7N 5E5 Canada

**Keywords:** Breast cancer cell lines, Signaling pathway activation, Tumorigenic, Metastatic, Mutations, Protein expression

## Abstract

**Background:**

Breast cancer cell lines are frequently used as model systems to study the cellular properties and biology of breast cancer. Our objective was to characterize a large, commonly employed panel of breast cancer cell lines obtained from the American Type Culture Collection (ATCC 30-4500 K) to enable researchers to make more informed decisions in selecting cell lines for specific studies. Information about these cell lines was obtained from a wide variety of sources. In addition, new information about cellular pathways that are activated within each cell line was generated.

**Methods:**

We determined key protein expression data using immunoblot analyses. In addition, two analyses on serum-starved cells were carried out to identify cellular proteins and pathways that are activated in these cells. These analyses were performed using a commercial PathScan array and a novel and more extensive phosphopeptide-based kinome analysis that queries 1290 phosphorylation events in major signaling pathways. Data about this panel of breast cancer cell lines was also accessed from several online sources, compiled and summarized for the following areas: molecular classification, mRNA expression, mutational status of key proteins and other possible cancer-associated mutations, and the tumorigenic and metastatic capacity in mouse xenograft models of breast cancer.

**Results:**

The cell lines that were characterized included 10 estrogen receptor (ER)-positive, 12 human epidermal growth factor receptor 2 (HER2)-amplified and 18 triple negative breast cancer cell lines, in addition to 4 non-tumorigenic breast cell lines. Within each subtype, there was significant genetic heterogeneity that could impact both the selection of model cell lines and the interpretation of the results obtained. To capture the net activation of key signaling pathways as a result of these mutational combinations, profiled pathway activation status was examined. This provided further clarity for which cell lines were particularly deregulated in common or unique ways.

**Conclusions:**

These two new kinase or “Kin-OMIC” analyses add another dimension of important data about these frequently used breast cancer cell lines. This will assist researchers in selecting the most appropriate cell lines to use for breast cancer studies and provide context for the interpretation of the emerging results.

**Electronic supplementary material:**

The online version of this article (doi:10.1186/s13058-017-0855-0) contains supplementary material, which is available to authorized users.

## Background

The general subtyping of breast cancer in the clinic is based on the expression of three main types of receptor: estrogen receptor (ER), progesterone receptor (PR) and the human epidermal growth factor receptor 2 (HER2, also known as ErbB2). ER+ breast cancers (60% of breast cancers) express ER ± PR and can be treated with anti-estrogens, such as tamoxifen, or aromatase inhibitors to block the generation of estrogen [1]. HER2 breast cancers (10 − 15% of breast cancers) overexpress HER2 receptors and can thus benefit from anti-HER2 antibodies, such as trastusumab, which block cell surface receptor dimerization with other family members and the activation of downstream signaling pathways. Triple negative breast cancers (TNBC; 15–20% of breast cancers) lack ER and PR, and do not overexpress HER2. As such, TNBC have no targeted therapies, are currently treated with chemotherapy, and have the poorest prognosis [1, 2].

Additional gene expression analyses have allowed for a more refined subgrouping of these subtypes that often helps to predict treatment responsiveness [3–10]. Luminal A cancers (ER+, PR±, HER2-) typically have a low proliferative capacity (low Ki67, a proliferative marker) and are often responsive to both endocrine and chemotherapy treatments [10]. Luminal B cancers (ER+, PR±, HER2+) have high Ki67 expression and usually respond to both endocrine and trastusumab treatments, with variable responses to chemotherapy [10]. HER2-amplified breast cancers (ER-, PR-, HER2+) overexpress high levels of HER2, have high Ki67 expression, and are responsive to trastusumb therapy and chemotherapy [10]. Basal A, also called “basal” cancers (ER-, PR-, HER2-) have high Ki67 expression, typically express epidermal growth factor receptor (EGFR+) and/or cytokeratin 5/6, and frequently respond to chemotherapy [10]. Basal B or claudin-low cancers are also ER-, PR-, HER2-, have low Ki67, E-cadherin, and claudin-3/4/7 expression, and have an intermediate response to chemotherapy [10].

There are numerous additional genes with variable expression levels and/or mutations within breast cancer cells, which contribute to a diverse genetic background that could influence therapeutic responses. As such, it is important to appropriately select breast cancer cell lines that accurately reflect this diversity when carrying out breast cancer studies. Further, if the molecular characteristics of the breast cancer cell lines are known, their ability to influence the results of experiments can be more effectively considered.

In this report, information was compiled from a variety of sources about a large panel of breast cancer cell lines that included the mutational status and mRNA expression of many important genes, and the tumorigenicity and metastatic properties in mouse xenograft models. Protein expression levels were examined for the corresponding gene products and we noted that these did not always correspond to the mRNA levels. In addition, lysates from serum-starved cells were used to carry out two types of pathway activation analyses to assess the activation of various signaling pathways within each cell line.

## Methods

### Cell culture

A panel containing 40 breast cancer cell lines and 4 non-tumorigenic breast cell lines was obtained from the American Type Culture Collection (ATCC, Manassas, Virginia, USA 30-4500 K; [11]). Cells were cultured according to ATCC recommendations for fewer than six months from the time of resuscitation. All cell lines were authenticated by the supplier.

### Immunoblot analysis

Protein expression in the breast cancer cell lines was quantified by immunoblot analysis as previously described [12]. Briefly, SDS-PAGE was performed loading an equal amount of total protein from cell lysates in each lane as determined by Lowry assay (Sigma Aldrich, Oakville, ON, Canada TP0300). Samples were transferred to nitrocellulose membranes and probed with primary antibodies. Antibodies were obtained from Santa Cruz Biotechnology (Dallas, TX, USA) for ER alpha (sc-8002), PR (sc-538), HER2 (sc-284), phosphatase and tensin homolog (PTEN) (sc-7974) and glyceraldehyde-3-phosphate dehydrogenase (GAPDH) (sc-25778), and from Cell Signaling Technology (Danvers, MA, USA) for p110α (4249) and p110β (3011). Additional primary antibodies included: EGFR (BD Biosciences, Mississauga, ON, Canada; 610017), p53 (ProteinTech, Rosemont, IL, USA; 10442-1-AP) and p85α (Cedarlane, Burlington, ON, Canada; 05-212). Blots were then probed with infrared 680 nm or 800 nm dye-tagged secondary antibodies (LI-COR Biosciences, Lincoln, NE, USA; 200 ng/ml) and were imaged with the Odyssey Infrared Imaging System (LI-COR Biosciences, Lincoln, NE, USA). The four gels required for each experiment were resolved, transferred, probed, washed, scanned and processed together to minimize technical artifacts. Blots shown are representative of at least three independent experiments, each using a fresh cell lysate.

### Database analyses

The ATCC website (http://www.ATCC.org; accessed May 14, 2015) provided basic information regarding the cell lines, including the sources used to generate the cell line, the type of breast cancer, and in some instances, the molecular classification or subtype, and partial data on the expression or absence of some genes. The catalogue of somatic mutations in cancer (COSMIC; http://cancer.sanger.ac.uk/cell_lines) database of cell line mutations was accessed January 30, 2016 (version v75). For Additional file 1: Table S3, the list of mutated genes was filtered using the online tools to provide only the cancer genes considered to be pathogenic. The cancer cell line encyclopedia (CCLE; https://www.broadinstitute.org/software/cprg/?q=node/11; 2012 September version [13]) dataset containing the robust multi-array average (RMA) and quantile-normalized mRNA expression was used. The values for normalized mRNA expression were then divided into four groups for each gene product as follows: ER and PR (>9 = +++, 7-8.9 = ++, 5-6.9 = +, <5 = -); HER2/ErbB2 (>10 = +++, 9-9.9 = ++, 8-8.9 = +, <8 = -); EGFR, TP53, BRCA1, BRCA2, p110α, p110β, p85α and PTEN (>9 = +++, 8-8.9 = ++, 7-7.9 = +, <7 = -).

### PathScan analysis

Cells were cultured under serum-starvation conditions (0.5% fetal bovine serum (FBS) containing medium) for 24 hours to analyze the signaling proteins/pathways activated within these cell lines. Cells were lysed and used to probe a PathScan RTK Signaling Antibody Array (Cell Signaling Technology, distributed by New England Biolabs, Whitby, ON, Canada; 7949) using reagents provided within the kit and according to the supplier’s instructions. These slide-based antibody arrays enable the simultaneous quantification of the extent of phosphorylation of 28 receptor tyrosine kinases and 11 key signaling proteins. The nitrocellulose-coated glass slides have highly specific capture antibodies that selectively bind target proteins within the cell lysate. A biotinylated detection antibody mixture and streptavidin-linked DyLight 680 molecule allow for the detection of bound protein using the Odyssey Infrared Imaging System (LI-COR Biosciences, Lincoln, NE, USA). Quantification was carried out using Odyssey V3.0 software. The raw intensity data for each target (mean of duplicate target measurements on each slide) had the background subtracted (mean of the 2 negative control spots), and was reported as a percentage of positive control spots (mean of 10 positive control spots). Data for the targets are reported for each cell line as the mean ± standard deviation of at least two (but for most three) independent experiments containing duplicate measurements, each using a fresh lysate. Hierarchical clustering was performed on both the phosphoproteins and cell lines using Ward's method (minimize cluster variance) with the Euclidean distance set as the distance metric. The heatmap and dendograms were generated using the Matplotlib and SciPy libraries for Python.

### Kinome analysis

A customized peptide array was developed to consider cancer-associated pathways, proteins and phosphorylation events. Phosphorylation events represented on the array were selected from databases of experimentally defined phosphorylation events and from those predicted by the software program DAPPLE2 [14]. Additional peptide substrates were included to represent proteins, and their associated phosphorylation events, that were shown in the literature to be differentially regulated in a number of cancers including renal cell carcinoma, pancreatic cancer, and prostate cancer [15–17]. Major signaling pathways involved in proliferation, metabolism, and apoptosis were also included on the array in order to give a general overview of the cell signaling patterns. In total, 1290 15-mer peptides were rationally selected for the array.

Design, construction, and application of the peptide arrays were based upon a previously reported protocol with modifications [18, 19]. Briefly, cells were serum-starved by growing in 0.5% FBS for 24 hours and 10 × 10^6^ cells were collected, pelleted, and lysed by addition of 100 μl of lysis buffer (20 mM Tris-HCl, pH 7.5, 150 mM NaCl, 1 mM EDTA, 1 mM EGTA, 1% Triton X-100, 2.5 mM sodium pyrophosphate, 1 mM sodium orthovanadate, 1 mM sodium fluoride, 1 μg/ml leupeptin, 1 μg/ml aprotinin, and 1 mM phenylmethylsulfonyl fluoride) (all from Sigma-Aldrich unless indicated). Cells were incubated on ice for 10 minutes and spun in a microcentrifuge for 10 minutes at 4 °C. A 70-μl aliquot of this supernatant was mixed with 10 μl of activation mix (50% glycerol, 500 μM ATP (New England BioLabs, Pickering, ON, Canada), 60 mM MgCl_2_, 0.05% (v/v) Brij 35, 0.25 mg/ml bovine serum albumin) and incubated on the array for 2 hours at 37 °C. Arrays were then washed with phosphate-buffered saline (PBS) + 1% Triton X-100. Slides were submerged in phosphospecific fluorescent ProQ Diamond Phosphoprotein Stain (Invitrogen, ThermoFisher, Burlington, ON, Canada) with agitation for 1 hour. Arrays were then washed three times in destaining solution containing 20% acetonitrile (EMD Biosciences, VWR Distributor, Mississauga, ON, Canada) and 50 mM sodium acetate at pH 4.0 for 10 minutes. A final wash was done with distilled deionized water. Arrays were air dried for 20 minutes and then centrifuged at 300 × *g* for 2 minutes to remove any remaining moisture. Arrays were analyzed using a GenePix Professional 4200A microarray scanner (MDS Analytical Technologies, Toronto, ON, Canada) at 532 to 560 nm with a 580-nm filter to detect dye fluorescence. Images were collected using GenePix software (version 6.0) and the spot intensity signal was collected as the mean of pixel intensity using local feature background intensity calculation with the default scanner saturation level.

All data processing and analysis was done using the Platform for Intelligent, Integrated Kinome Analysis (PIIKA) software [20], which is freely available for non-commercial use at http://saphire.usask.ca/saphire/piika. For each peptide within a given array, the chi-square test was performed to determine whether the degree of variability among the technical replicates for that peptide was greater than would be expected by chance. Any peptide that had a *P* value according to the chi-square test of less than 0.01 was considered to be inconsistently phosphorylated among the technical replicates and was excluded from further analysis.

The preprocessed data were subjected to hierarchical clustering and principal component analysis (PCA) to cluster peptide response profiles across cell lines. For each of the 1290 peptides in a single sample and cell line, the average was taken over the nine replicates that are transformed through variance stabilization and normalization (VSN). For hierarchical clustering, each sample/cell line vector was considered a singleton (i.e., a cluster with a single element) at the initial stage of the clustering. The two most similar clusters were merged, and the distances between the newly merged clusters and the remaining clusters were updated, iteratively. The method, as described by Eisen et al. [21], used the following calculation: average linkage + (1 - Pearson correlation). The method takes the average over the merged (i.e., the most correlated) kinome profiles and updates the distances between the merged clusters and other clusters by recalculating the correlations between them.

InnateDB is a publicly available resource which, based on levels of either differential expression or phosphorylation, predicts biological pathways based on experiment fold change data sets [22]. Pathways were assigned a probability value (*P*) based on the number of proteins present for a particular pathway and the degree to which they were differentially expressed or modified relative to a control condition.

For hierarchical clustering of kinome profiles, the distance metric used was (1 - Pearson correlation), while McQuitty linkage was used as the linkage method. Colors indicate the average (over nine intra-array replicates) normalized phosphorylation intensity of each target, with red indicating increased phosphorylation and green indicating decreased phosphorylation. The intensity of the color corresponds to the degree of increase or decrease [23].

## Results and discussion

### Molecular features and tumorigenicity of breast cancer cell lines

We have compiled the currently available data and carried out further analyses to better classify and characterize a panel of 40 breast cancer cell lines compared to 4 non-tumorigenic control breast cell lines. Several studies using large groups of breast cancer cell lines have evaluated gene expression data to molecularly classify each cell line from general subtypes of normal, ER+, HER2-amplified, and TNBC and into further subgroups of luminal A, luminal B, HER2-amplified, basal A, and basal B (also known as claudin-low) [3, 4, 6, 7]. We have compiled these data together with those from the ATCC [11], the commercial source of these cell lines (Additional file 2: Table S1). In addition, the mutational status of key genes was obtained from COSMIC [24] and mRNA expression levels from the CCLE [13]. Furthermore, protein expression levels for ER, PR, HER2, EGFR, and p53 were determined within this large group of cell lines using an immunoblot analysis (Additional file 2: Table S1 and Additional file 3: Figure S1). There have been conflicting reports as to whether the MDA-MB-453 cell line is HER2-amplified or TNBC/basal A, and by extension, MDA-kb2 that was derived from MDA-MB-453. These inconsistencies likely resulted from the high HER2 mRNA levels, yet relatively low HER2 protein expression. Based on the HER2 protein expression observed in the immunoblots (Additional file 3: Figure S1), we classified MDA-MB-453 and MDA-kb2 as TNBC/basal A. Several cell lines show a similar discrepancy in PR levels with relatively high mRNA expression, yet little or no PR protein, including HCC1428, MCF7, UACC812, and ZR-75-1. We also noted that two cell lines had higher levels of protein expression than would be predicted from the mRNA levels, including very high ER expression in HCC1500 cells and some EGFR expression in HCC38 cells. The general disconnect between mRNA and protein expression has been described in previous reports [25, 26].

In some cell lines, and in contrast to the wild-type status and positive p53 mRNA expression for HCC1428, MCF7 and ZR-75-30 cells, there was minimal detectable expression of p53 protein (Additional file 2: Table S1). There were also several cell lines in which p53 protein levels were much higher than the corresponding mRNA levels would predict, including for HCC1937 (WT), HCC38 (R273L mutation), HCC2157 (R248W), MDA-MB-231 (R280K), and SK-BR-3 (R175H). The R175H mutation in p53 is present in both AU565 and also SK-BR-3 cells with very different levels of p53 protein expression. This result suggests that protein stability is not affected by the R175H mutation and instead other factors are influencing p53 protein expression. Thus, caution should be exercised when inferring protein expression based on mRNA levels, since in several instances they do not correspond.

The mutational status and mRNA expression levels for BRCA1 and BRCA2 were also collected (Additional file 2: Table S1). Several cell lines with the wild-type *BRCA1* gene did not express BRCA1 mRNA, including AU565, HCC38, HCC2218, Hs578T and MDA-MB-134-VI. For BRCA2, most of the cell lines contained a wild-type gene, and yet most lacked detectable levels of BRCA2 mRNA with the exception of HCC1187 and HCC1937. These results are consistent with reports of epigenetic mechanisms that downregulate gene expression in breast cancer, particularly that of BRCA1 and BRCA2 [27–29].

We further focused on the analysis of key phosphatidylinositol 3-kinase (PI3K) pathway genes that when mutated or differentially expressed would promote PI3K pathway activation, which is critically important in tumorigenesis. As described, mutational data were obtained from the COSMIC database, mRNA expression from the CCLE, and an immunoblot analysis for protein expression was performed (Additional file 4: Table S2 and Additional file 5: Figure S2). Consistent with previous observations about breast cancer cells, PI3K pathway activating events are frequent, particularly the loss of PTEN expression and the presence of activating mutations in the PIK3CA gene encoding p110α, usually at the hot spot sites (E545K, E542K, and H1047R) [9]. In addition, reductions in p85α expression were fairly common and the more rare amplification of p110β, specifically at the level of protein expression, was observed. Each of these alterations has been shown to contribute to PI3K pathway activation [6, 30–35]. Several cell lines do not express p110α protein, which could reduce the net PI3K pathway signaling (Additional file 4: Table S2 and Additional file 5: Figure S2). The majority of the breast cancer cell lines analyzed showed activating alterations in one of these PI3K pathway components, but there were also several that showed two or more. Only three cell lines (HCC1187, MDA-MB-134-VI, and UACC812) showed no alterations in p110α, p110β, p85α, and PTEN, consistent with the high frequency of PI3K pathway activation events noted in breast cancers [9, 32].

To further aid in the selection of breast cancer cell line models, we also compiled additional cancer mutation data for each cell line, as determined using COSMIC, and their tumorigenic and metastatic potential in mouse xenograft models from various publications [36–61] (Additional file 1: Table S3). These mutational profiles provide additional insight into molecular alterations that could influence breast cancer cell behavior that may impact cell line choice or data interpretation. Our laboratory found this latter information particularly useful when selecting appropriate cell lines to study the role of CREB3L1 in breast cancer metastasis [62, 63].

### Characterization of activated receptor and signaling pathways

The analyses of the breast cancer cell lines and subtyping have focused primarily on the mutational status and expression (mRNA) of key genes, and to a lesser extent key proteins. This molecular information is fairly straightforward to obtain, but it does not provide a more integrated global readout of which cellular pathways are activated and to what extent. Therefore, we carried out two analyses to assess pathway activation within this panel of breast cancer cell lines.

To determine the key receptors and pathways that were activated within each of the cell lines as a result of the protein expression profiles and mutational status, cells were cultured for 24 hours in low serum (0.5% FBS) to minimize the impact of growth factor-mediated receptor and pathway activation. Two complementary analyses were then carried out. First, lysates were used to probe a commercially available PathScan RTK Signaling Antibody Array from Cell Signaling Technology. This analysis provided the activation status of 28 receptor tyrosine kinases and 11 downstream signaling proteins (Additional file 6: Table S4, Additional file 7: Figure S3 and Additional file 8: Figure S4).

**Fig. 1:**
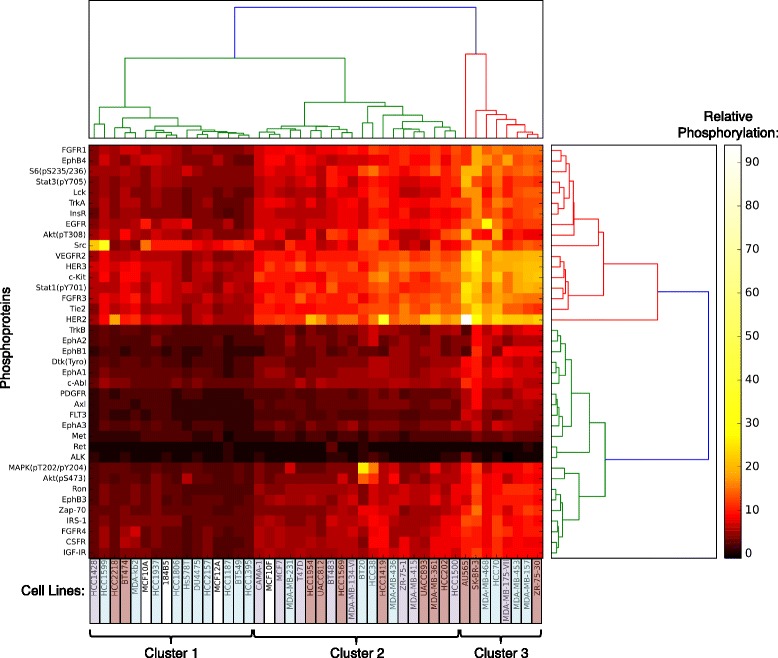
PathScan heatmap illustrating the clustering of breast cancer cell lines and target phosphorylation profiles. Hierarchical clustering was performed on both phosphoproteins and cell lines as detailed in "Methods". Cell line names have been color-coded as follows: estrogen receptor-positive (*purple*), human epidermal growth factor receptor 2 -amplified (*red*), and triple negative breast cancer (*blue*). Clusters of cell lines with similar phosphoprotein profiles have been numbered *1*−*3*, as indicated

We subsequently carried out a cluster analysis to identify groups of cell lines in which the activation of specific kinases gave similar phosphorylation profiles. We identified three main clusters (Fig. 1). Cluster 1 has the lowest levels of phosphorylation and contains many of the TNBC cell lines and non-tumorigenic breast lines, but also an ER+ (HCC1428) and two HER2-amplified (HCC2218 and BT474) cell lines. Cluster 2 has intermediate levels of phosphorylation and contains most of the ER+ cell lines (8/10), about half of the HER2-amplified lines (7/12), a few TNBC lines (4/18), and one non-tumorigenic breast line, MCF10F (Fig. 1). Cluster 3 has the most highly phosphorylated tyrosine kinases, including HER2, HER3, vascular endothelial growth factor receptor 2 (VEGFR2), c-Kit, Stat1, fibroblast growth factor receptor 3 (FGFR3), and tyrosine kinase with immunoglobulin-like and EGF-like domains 2 (Tie2), and includes several HER2-amplified cell lines (AU565, SK-BR-3, ZR-75-30). Surprisingly, this cluster also contains several TNBC cell lines (MDA-MB-468, HCC70, MDA-MB-157, MDA-MB-453), with a phosphorylation profile more similar to these HER2-amplified lines than the majority of the TNBC cell lines, and the ER+ cell line (MDA-MB-175-VII) (Fig. 1). This analysis suggests that these breast cancer cell lines can be grouped based on the phosphorylation profile, as an alternative to the standard criteria, depending on the studies to be performed. Protein phosphorylation status is important and could provide important mechanistic information for studies using these cell line models.

A second phosphoprotein activation analysis was carried out using serum-starved lysates to probe a custom-made cancer-specific peptide array of kinase target peptides, called a kinome array. This allowed for the analysis of a large number of kinase targets that may be active in this panel of breast cancer cell lines. The array consisted of 15-mer peptides (1290 total) corresponding to kinase substrates within major signaling pathways involved in key cellular processes, including cell proliferation, metabolism, and apoptosis (Additional file 9: Table S5). Phosphorylated kinome array peptides were quantified and reported relative to the mean signal of the corresponding peptide from four non-tumorigenic breast cell lines (Additional file 10: Table S6).

**Fig. 2:**
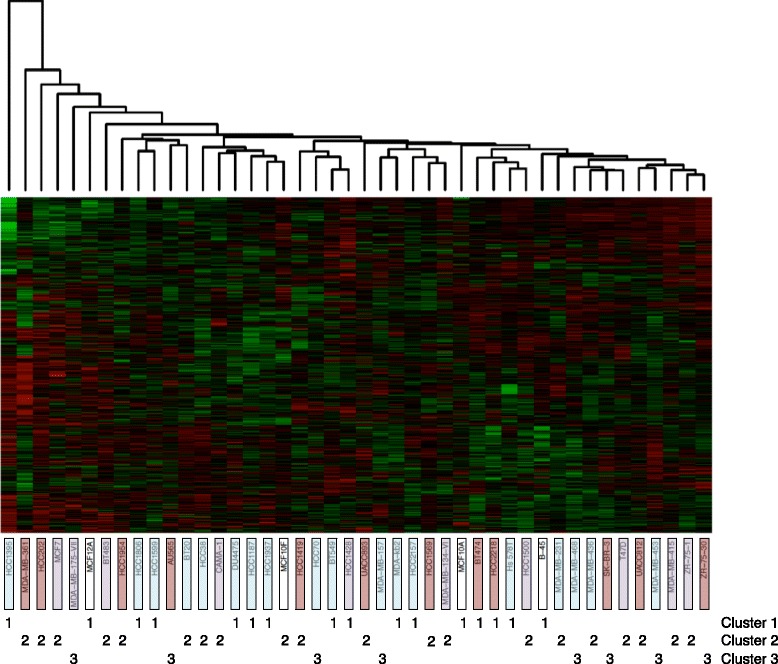
Kinome heatmap illustrating the clustering of breast cancer cell lines and target phosphorylation profiles. Hierarchical clustering was performed on both phosphoproteins and cell lines as detailed in “Methods”. *Rows* correspond to probes (phosphorylation targets), and *columns* correspond to samples. *Red* indicates increased phosphorylation and *green* indicates decreased phosphorylation, with the intensity of the color corresponding to the degree of increase or decrease. Cell line names have been color-coded as follows: estrogen receptor-positive (*purple*), human epidermal growth factor receptor 2-amplified (*red*), and triple negative breast cancer (*blue*). The cluster that each cell line was grouped into in "Fig. 1" has been indicated under each cell line name

We carried out a cluster analysis to identify degrees of similarity in the signaling profiles across cell lines (Fig. 2). In contrast to the PathScan data in which three distinct clusters of cell lines were identified based on 39 phosphoproteins, the kinome data provided more of a continuum in which adjacent cell lines were quite similar, but larger clusters of cell lines were not evident. No clustering of molecular subtypes of breast cancer was observed and the clustering noted in the PathScan data (Fig. 1) was not recapitulated in this more extensive analysis (Fig. 2). This is likely the result of the large number of phosphoproteins analyzed (i.e., 1290).

**Fig. 3:**
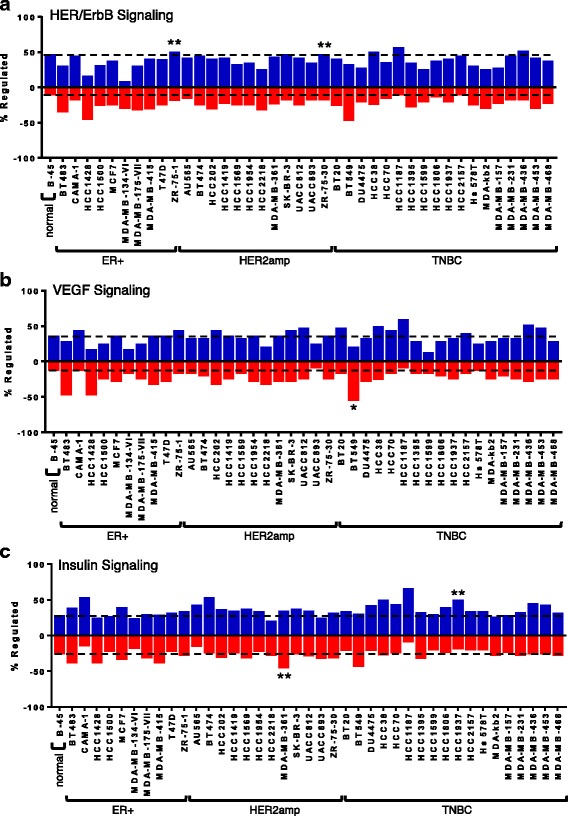
Kinome results of (**a**) HER/ErbB, (**b**) vascular endothelial growth factor (*VEGF*) and (**c**) insulin signaling pathways. For each cancer cell line, pathway overrepresentation analysis was performed using InnateDB relative to a control representing the averaged signaling profile of four non-cancer cell lines (184B5, MCF10A, MCF12A, and MCF10F; noted as normal, *B-45*). The percentage of peptides with increased or decreased phosphorylation is calculated relative to the total number of peptides on the array that are associated with the signaling pathway under consideration. Consideration is limited to peptides with consistent patterns of phosphorylation across the nine technical replicates (*P* < 0.05) and changes in the extent of phosphorylation are determined as differential phosphorylation (*P* < 0.05) relative to the control. The pathway overrepresentation analysis of InnateDB also provides *P* values for the activation or repression of the signaling pathway based on both the number of peptides that are consistently and differentially phosphorylated between the cancer cell line and the control cells and the magnitude of this phosphorylation difference. Cell lines with activation of the pathway are represented above the *vertical axis* (*blue*) or below for those with repression (*red*) (*P < 0.10; **P < 0.05). *Dashed lines* indicate the level of phosphorylation for the four averaged normal cell lines (i.e., B-45) to help identify differences for the cancer cell lines. *HER2amp* human epidermal growth factor receptor 2-amplified, *ER* estrogen receptor, *TNBC* triple negative breast cancer

**Fig. 4:**
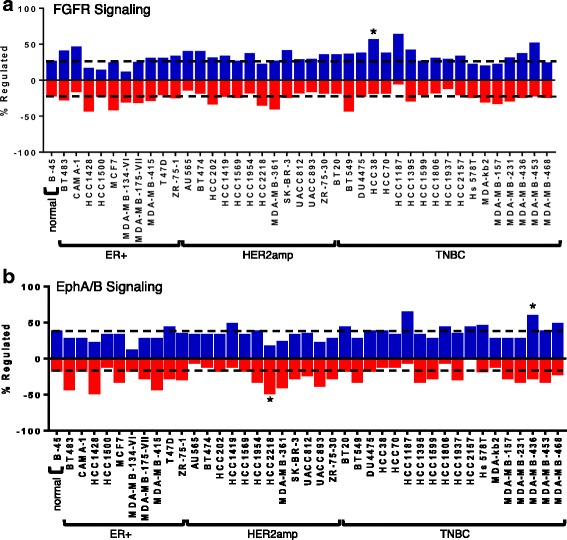
Kinome results for the (**a**) fibroblast growth factor receptor (*FGFR*) and (**b**) ephrin (EphA/B) signaling pathways. The analysis was carried out as detailed for “Fig. 3”. *HER2amp* human epidermal growth factor receptor 2-amplified, *ER* estrogen receptor, *TNBC* triple negative breast cancer

**Fig. 5:**
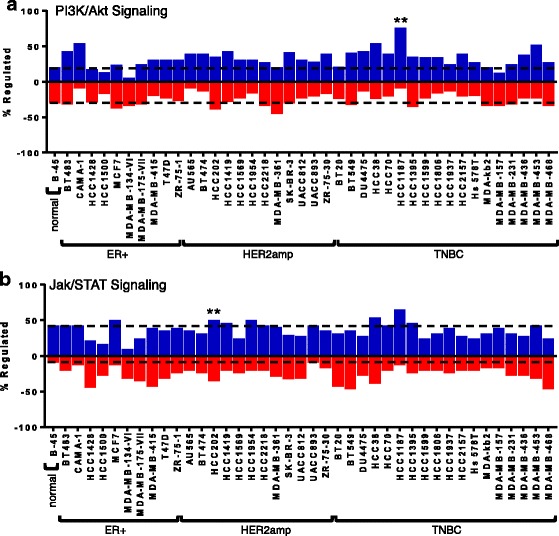
Kinome results for the (**a**) PI3K/Akt and (**b**) Jak/STAT signaling pathways. The analysis was carried out as detailed for “Fig. 3” *HER2amp* human epidermal growth factor receptor 2-amplified, *ER* estrogen receptor, *TNBC* triple negative breast cancer

The kinase substrates were also grouped into the major biological pathways in which they are known to play a role, realizing that many contribute to the regulation of several pathways (Additional file 11: Table S7). The number of peptides with increased or decreased phosphorylation was determined for each cell line relative to the mean of the control non-tumorigenic breast cell lines. Several pathways showed large differences between the breast cancer cell lines and the control breast cells and have been graphed to illustrate the fraction of the target peptides with upregulated phosphorylation and downregulated phosphorylation within the same pathway (Figs. 3, 4 and 5). This analysis takes into account each component in the pathway to determine the overall phosphorylation status of the pathway. The statistical analysis factors in the fold change in the phosphorylation of each target (Additional file 10: Table S6), for the determination of *P* values.

The HER/ErbB signaling pathway was found to be significantly activated in the ER+ cell line ZR-75-1, and in the HER2-amplified cell line ZR-75-30 (Fig. 3a). VEGF signaling had reduced activity in BT549 cells (Fig. 3b). Insulin signaling had reduced activity in MDA-MB-231 cells, but enhanced activity in HCC1937 cells (Fig. 3c). FGFR signaling was increased in HCC38 cells (Fig. 4a), whereas EphA/B signaling was decreased in HCC2218 cells and increased in MDA-MB-436 cells (Fig. 4b). PI3K/Akt signaling was enhanced in HCC1187 cells (Fig. 5a) and Jak/STAT signaling was activated in HCC202 cells (Fig. 5b). This analysis highlights the most prominent pathways that are altered within the cell lines.

More specific changes in the phosphorylation of individual targets can be mined from the complete dataset (Additional file 10: Table S6). There were some targets that were frequently substantially more phosphorylated or less phosphorylated across most cell lines regardless of the subtype of breast cancer. For example, one target that showed a general increase in phosphorylation in most cell lines was PPP1R12A (on S445) (Additional file 10: Table S6, row 936). When PPP1R12A is phosphorylated on S445 (by LATS) it dephosphorylates T210 of PLK1 in an attempt to inactivate this frequently overexpressed kinase involved in cell cycle progression [64, 65]. A second target with increased phosphorylation across most cell lines was STAT3 (on Y705) (Additional file 10: Table S6, row 1108). Phosphorylation of STAT on this site is important for cell migration, invasion and anchorage-independent growth [66].

In contrast, some targets such as IKKα (on T23) showed a decrease in phosphorylation in many cell lines (Additional file 10: Table S6, row 559). Akt phosphorylates T23 of IKKα to activate NF-κB and promote cell survival [67]. A second target with decreased phosphorylation in many cell lines was CDK1 (on T14) (Additional file 10: Table S6, row 270). Dephosphorylation of T14 releases its inhibition to activate CDK1 and promote cell cycle progression [68].

Several cell lines displayed a larger number of phosphorylated targets with substantial changes (Additional file 10: Table S6). These included: several ER+ (MCF7, HCC1428, and MDA-MB-175-VII), HER2-amplified (HCC202, HCC1419, and MDA-MB-231) and TNBC (HCC1395 and HCC38) cell lines. The data on these activated or inactivated signaling targets will be a valuable resource in the selection of cell lines and interpretation of data for breast cancer cell line studies.

## Conclusions

In this report a resource has been created that includes key cancer mutations, mRNA expression, and protein expression data for a large panel of 40 breast cancer cell lines, as compared to 4 non-tumorigenic control breast cell lines. The tumorigenic and metastatic properties in mouse xenograft models have also been compiled to aid in the selection of breast cancer models to study these processes. Important new information about the cellular proteins and pathways active within these cell lines has also been evaluated to facilitate both the choice of the best cell lines for a particular study, as well as to aid in the interpretation of experimental observations by providing a context for the discussion of the results obtained. This will be a valuable resource for the breast cancer research community.

## Additional files

Additional file 1: **Table S3.** Mutations in the breast cancer cell lines plus their tumorigenic and metastatic ability in mouse xenografts. (XLSX 13 kb)

Additional file 2: **Table S1.** Basic expression profile and molecular classification of a panel of breast cancer cell lines. (XLSX 19 kb)

Additional file 3: **Figure S1.** Protein expression analysis to aid in the molecular classification of breast cancer cell lines. Cell lysates containing equivalent amounts of total cell protein from the indicated non-tumorigenic breast (184B5, MCF10A, MCF10F, MCF12A) and breast cancer cell lines were probed with the indicated antibodies and GAPDH (loading control). The amount of total protein loaded per lane for each set of blots was as follows: ER (50 μg), PR (50 μg; PR-A is 81 kDa and PR-B is 116 kDa), HER2 (10 μg), EGFR (50 μg), p53 (50 μg), and GAPDH (50 μg). The molecular weight of each protein is indicated. (PDF 259 kb)

Additional file 4: **Table S2.** Mutations, mRNA and protein expression for PI3K pathway components in breast cancer cell lines. (XLSX 15 kb)

Additional file 5: **Figure S2.** PI3K pathway protein expression analysis for breast cancer cell lines. Cell lysates containing equivalent amounts of total cell protein from the indicated non-tumorigenic breast (184B5, MCF10A, MCF10F, MCF12A) and breast cancer cell lines were probed with the indicated antibodies and GAPDH (loading control). The amount of total protein loaded per lane for each set of blots was as follows: p110α (50 μg), p110β (50 μg), p85α (25 μg), PTEN (50 μg), and GAPDH (50 μg). The molecular weight of each protein is indicated. (PDF 234 kb)

Additional file 6: **Table S4.** Pathscan analysis of receptors and pathways intrinsically activated in a panel of breast cancer cell lines. (XLSX 67 kb)

Additional file 7: **Figure S3.** Downstream signaling pathway activation in breast cancer cell lines. Lysates from the indicated breast cancer cell lines that had been grown under serum-starved conditions were used to probe a PathScan array to detect intrinsic activation of the indicated proteins using pan-pTyr, or the specified phosphospecific antibody. The schematic image of the array was reproduced courtesy of Cell Signaling Technology, Inc. (www.cellsignal.com). (PDF 390 kb)

Additional file 8: **Figure S4.** Downstream signaling pathway activation in additional breast cancer cell lines. Lysates from the indicated breast cancer cell lines that had been grown under serum-starved conditions were used to probe a PathScan array to detect activation of the indicated proteins using pan-pTyr, or the specified phosphospecific antibody. The schematic image of the array was reproduced courtesy of Cell Signaling Technology, Inc. (www.cellsignal.com). (PDF 398 kb)

Additional file 9: **Table S5.** Kinome peptide list. List of protein substrates from which peptides were derived, the 15-mer peptide sequence, and the specific amino acid(s) with phosphorylation that was detected if phosphorylated by a kinase from the cell lysate. The amino acid phosphorylation site numbering is for the human protein although some of the phosphorylations were originally identified at the equivalent sites in other species. (XLSX 106 kb)

Additional file 10: **Table S6.** Complete kinome data for all peptide substrates and all breast cell lines evaluated. Fold change is given for the phosphorylation of each peptide substrate, relative to the mean of four non-tumorigenic breast cell lines (184B5, MCF10A, MCF10F, MCF12A). Site refers to the amino acid residue in the human protein. (XLSX 443 kb)

Additional file 11: **Table S7.** Kinome peptides in array, grouped according to the major biological pathways to which they contribute. (XLSX 38 kb)

## Abbreviations

ATCC: American Type Culture Collection
CCLE: Cancer Cell Line Encyclopedia
COSMIC: Catalogue of Somatic Mutations in Cancer
CREB3L1: cAMP responsive element binding protein 3-like 1
EGFR: Epidermal growth factor receptor
EphA/B: Ephrin A/B
ER: Estrogen receptor
FBS: Fetal bovine serum
FGFR: Fibroblast growth factor receptor
GAPDH: Glyceraldehyde-3-phosphate dehydrogenase
HER2: Human epidermal growth factor receptor 2
PI3K: Phosphatidylinositol 3-kinase
PR: Progesterone receptor
PTEN: Phosphatase and tensin homolog
RMA: Robust multi-array average
Tie2: Tyrosine kinase with immunoglobulin-like and EGF-like domains 2
TNBC: Triple negative breast cancer
VEGFR: Vascular endothelial growth factor receptor
