# Supplementary figures and images for: Molecular characterization of breast cancer cell lines through multiple omic approaches
Source: Breast Cancer Res. 2017 Jun 5;19:65. doi: 10.1186/s13058-017-0855-0 (PMC5460504; doi:10.1186/s13058-017-0855-0)

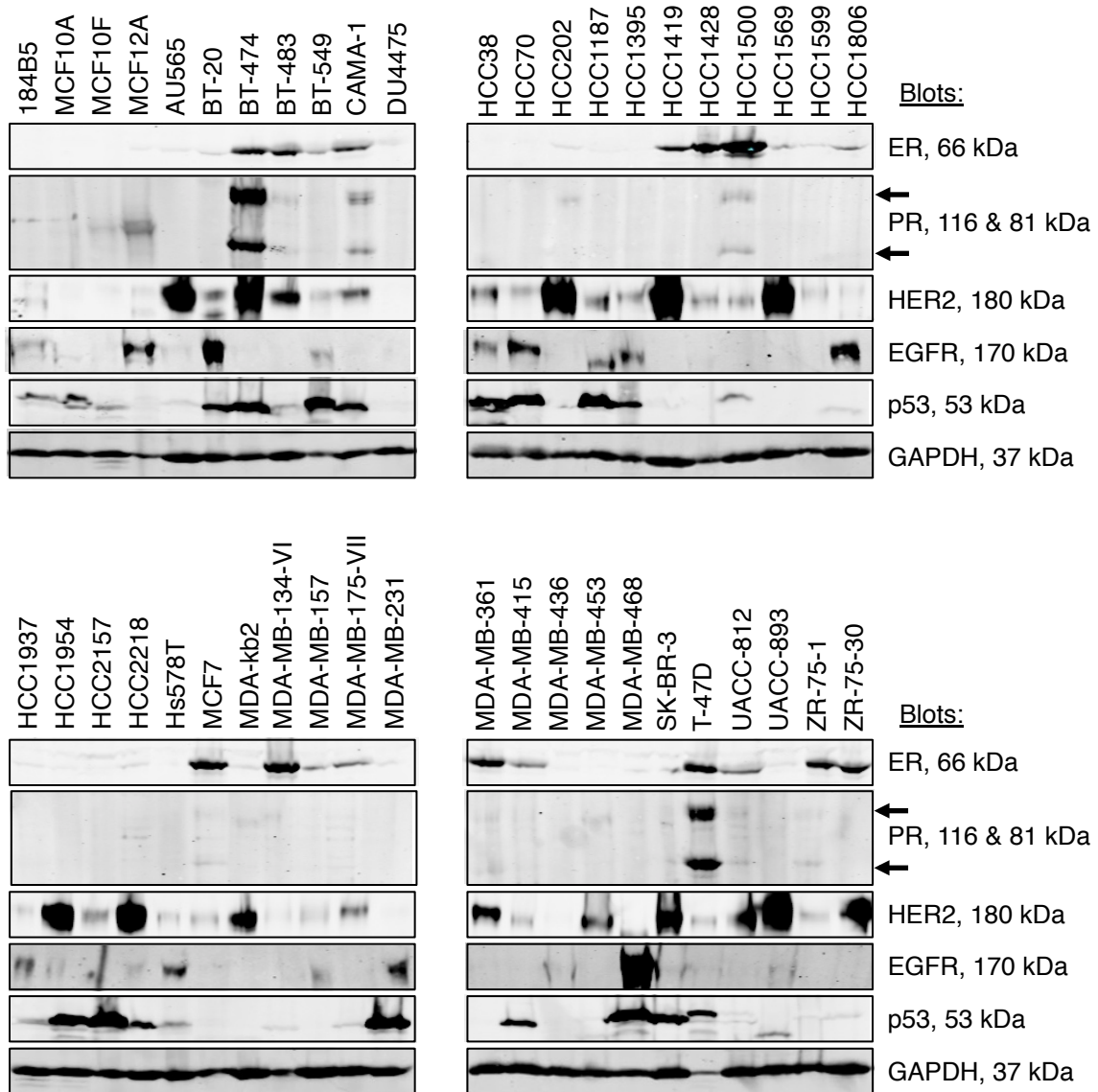

Supplement: Supplementary file 3 — Figure S1. Protein expression analysis to aid in the molecular classification of breast cancer cell lines. Cell lysates containing equivalent amounts of total cell protein from the indicated non-tumorigenic breast (184B5, MCF10A, MCF10F, MCF12A) and breast cancer cell lines were probed with the indicated antibodies and GAPDH (loading control). The amount of total protein loaded per lane for each set of blots was as follows: ER (50 μg), PR (50 μg; PR-A is 81 kDa and PR-B is 116 kDa), HER2 (10 μg), EGFR (50 μg), p53 (50 μg), and GAPDH (50 μg). The molecular weight of each protein is indicated. (PDF 259 kb) [file 13058_2017_855_MOESM3_ESM.pdf]

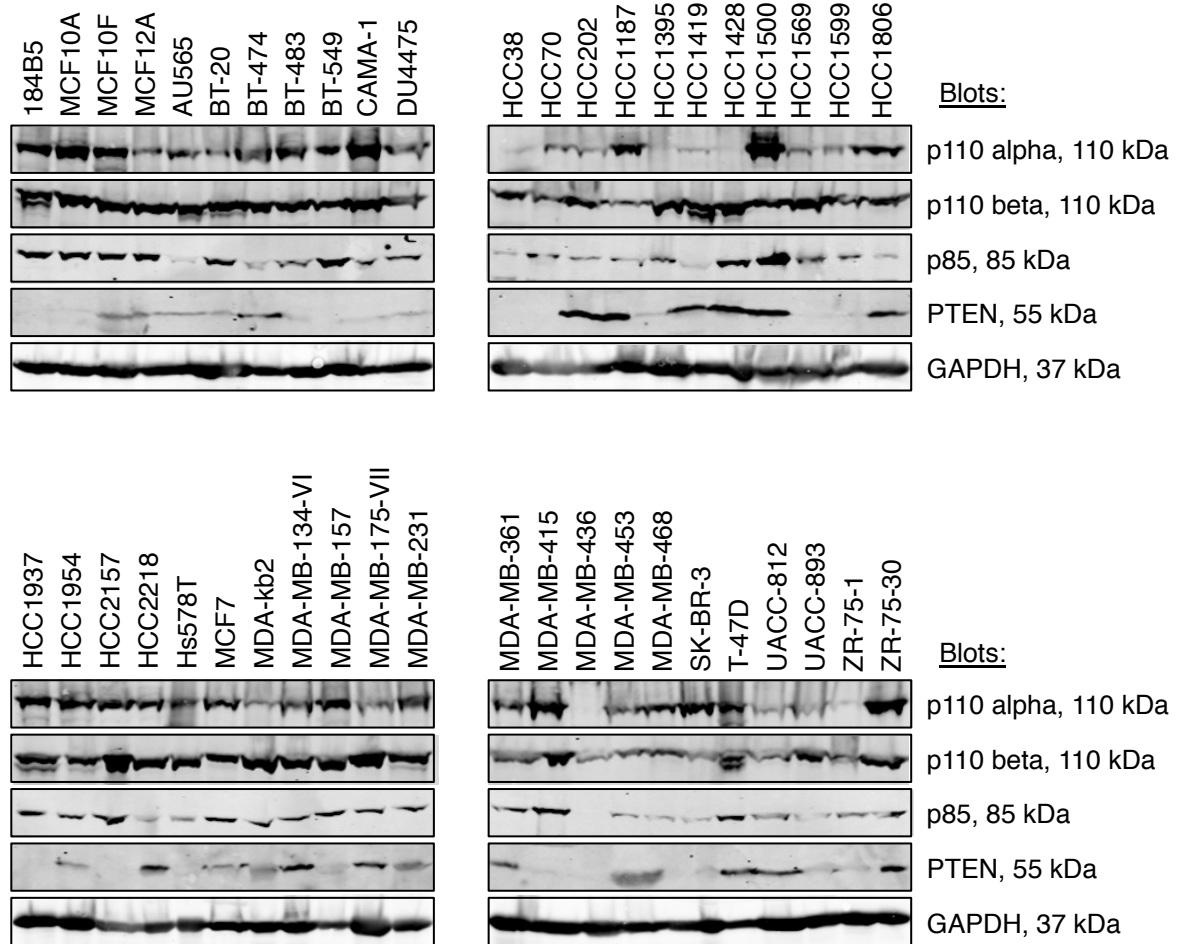

Supplement: Supplementary file 5 — Figure S2. PI3K pathway protein expression analysis for breast cancer cell lines. Cell lysates containing equivalent amounts of total cell protein from the indicated non-tumorigenic breast (184B5, MCF10A, MCF10F, MCF12A) and breast cancer cell lines were probed with the indicated antibodies and GAPDH (loading control). The amount of total protein loaded per lane for each set of blots was as follows: p110α (50 μg), p110β (50 μg), p85α (25 μg), PTEN (50 μg), and GAPDH (50 μg). The molecular weight of each protein is indicated. (PDF 234 kb) [file 13058_2017_855_MOESM5_ESM.pdf]

Additional file 7: Figure S3. Smith *et al.*

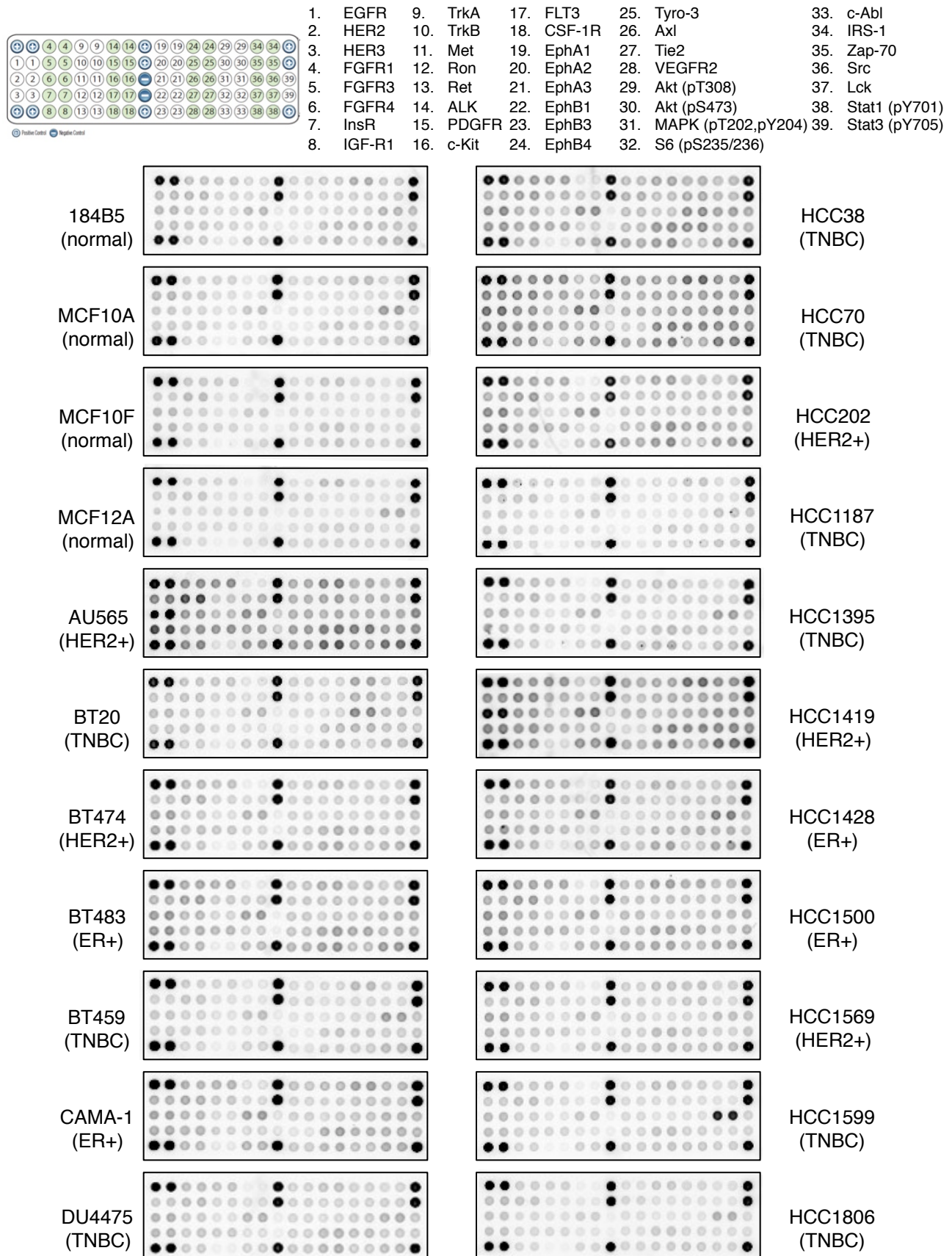

Supplement: Supplementary file 7 — Figure S3. Downstream signaling pathway activation in breast cancer cell lines. Lysates from the indicated breast cancer cell lines that had been grown under serum-starved conditions were used to probe a PathScan array to detect intrinsic activation of the indicated proteins using pan-pTyr, or the specified phosphospecific antibody. The schematic image of the array was reproduced courtesy of Cell Signaling Technology, Inc. (www.cellsignal.com). (PDF 390 kb) [file 13058_2017_855_MOESM7_ESM.pdf]

Additional file 8: Figure S4. Smith *et al.*

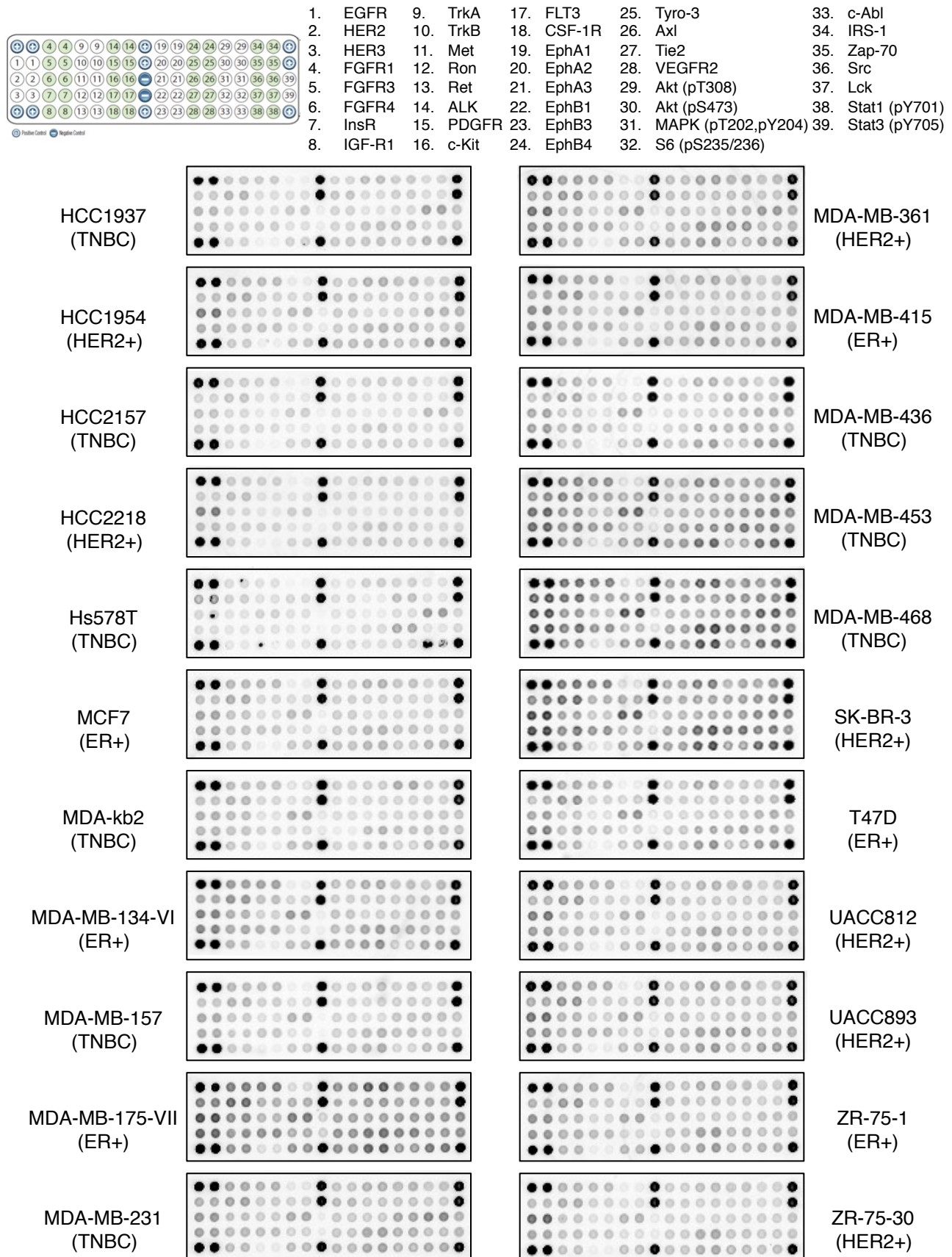

Supplement: Supplementary file 8 — Figure S4. Downstream signaling pathway activation in additional breast cancer cell lines. Lysates from the indicated breast cancer cell lines that had been grown under serum-starved conditions were used to probe a PathScan array to detect activation of the indicated proteins using pan-pTyr, or the specified phosphospecific antibody. The schematic image of the array was reproduced courtesy of Cell Signaling Technology, Inc. (www.cellsignal.com). (PDF 398 kb) [file 13058_2017_855_MOESM8_ESM.pdf]
